# Novel binding partners for Prenylated Rab Acceptor 1 identified by a split-ubiquitin yeast two-hybrid screen

**DOI:** 10.1186/s13104-019-4219-y

**Published:** 2019-03-29

**Authors:** Ameair Abu Irqeba, Judith Mosinger Ogilvie

**Affiliations:** Biology Department, Saint Louis University, Macelwane Hall, 3507 Laclede Ave, St. Louis, MO 63103 USA

**Keywords:** PRA1, Rabac1, Yip3, Split-ubiquitin yeast two-hybrid screen, Photoreceptor, Mouse, Protein interaction

## Abstract

**Objective:**

Prenylated Rab Acceptor 1 (PRA1) is a transmembrane protein localized to the early secretory pathway. It has been found to interact with an array of Rab GTPases, leading to its hypothesized function in the recycling of Rab GTPases. However, all previous strategies used to screen for novel interacting partners have utilized a classic yeast two-hybrid approach that requires both bait and its potential binding partners to be cytosolic proteins. In the split-ubiquitin yeast two-hybrid screen, a protein interaction leads to the re-constitution of ubiquitin, which is followed by proteolytic release of a transcription activator that migrates to the nucleus alone. This allows for bait and/or prey to be integral membrane protein(s). To better understand the in vivo function of PRA1, we took an unbiased approach that screened PRA1 against a normalized mouse neuronal cDNA library using this variant of the classic screening strategy.

**Results:**

We report 41 previously unidentified potential PRA1 binding partners revealed by this screen and validate the screen by confirming three of these interactions using a bi-molecular fluorescence complementation assay in mammalian cells. The identified proteins reside throughout the secretory pathway and are both membrane-bound and cytosolic in their identity, suggesting alternative functions for PRA1.

## Introduction

Prenylated Rab Acceptor 1 (PRA1/Rabac1) is a ubiquitously expressed and highly conserved protein, a fragment of which was first identified as a Rab6 binding partner in a yeast two-hybrid screen [1]. The full-length open reading frame was further isolated in other yeast two-hybrid screens where Rab GTPase family members were used as bait [2–4]. This interaction was later found to be prenylation-dependent [5]. Functionally, this potential interaction, along with other in vitro evidence, has led to the suggestion that PRA1 plays a role in the recycling of Rab GTPases [6–8].

Recently, we completed a microarray analysis of retinal gene expression in the *rd1* mouse model of retinitis pigmentosa and found that PRA1 is significantly down-regulated beginning at postnatal day 2 (P2), well before the onset of photoreceptor degeneration [9]. Consistent with our observations in *rd1* photoreceptors, knock-down of PRA1 in HeLa cells alters both ER and Golgi morphology [10]. In addition, other reports indicate that down-regulation of PRA1 is not directly linked to erroneous trafficking of Rab GTPases [11, 12], which suggests that PRA1 may have other unidentified functions necessary for development and survival of photoreceptors and other cells [13].

To gain further insight into its true role in mammalian cells, we screened for novel PRA1 binding proteins. Previous studies targeted their screening approach to Rab GTPases and soluble proteins, thereby limiting identification of many other potential PRA1 binding partners. PRA1 contains four extensively hydrophobic transmembrane alpha-helices that would prevent nuclear translocation of any potential interacting complexes that formed in a classic yeast two-hybrid screen [14–16]. Using a split-ubiquitin yeast two-hybrid screen, we have identified 41 novel PRA1 binding partners and further validated these results by confirming three interactions in mammalian cells. These findings will promote further understanding of the functional role of PRA1 in vivo.

## Main text

### Methods

#### Split-ubiquitin yeast two-hybrid screen

The DUALmembrane starter kit (cat# P01201) was purchased from Dualsystems Biotech (Schlieren, Switzerland). The kit included the DSY Yeast transformation kit (cat# P01003), NMY51 Yeast reporter strain (cat# P04005), and a normalized adult mouse brain NUBG-X cDNA library (cat# P12303). An explicit, step-by-step protocol for the screening strategy used here has previously been published [17], and is described below.

All cloning reagents were purchased from New England Biolabs (Ipswitch, MA). Mouse PRA1 cDNA was purchased from Origene (Rockville, MD). Using the *Sfi*I restriction enzyme sites, the full length PRA1 open reading frame was cloned into the supplied pBT3-N vector, which was engineered to fuse LexA-VP16-CUB (CUB: amino acids 34–76 of ubiquitin) to the PRA1 cytosolic N-terminus. This construct was then sequence verified via Sanger sequencing by Eurofins (Louisville, KY). A normalized adult mouse neuronal cDNA library fused to NUB (amino acids 1–38 of ubiquitin) was used in this screen. The provided quality control data indicate that the library contains 2.2 × 10^6^ independent clones, average insert size is 1.1 kb (with a range of 0.6–10 kb), and 95%+ of vectors contain an insert. The NMY51 strain was used during the screening process (*MATa his3Δ200 trp1*-*901 leu2*-*3, 112 ade2 LYS2::(lexAop)*_*4*_-*HIS3 ura3::(lexAop)*_*8*_-*lacZ (lexAop)*_*8*_-*ADE2 GAL4*). Plasmids were introduced into *Saccharomyces cerevisiae* yeast via LiOAc/PEG transformation. NMY51 was initially streaked on YPD media and SD-drop out media was used where required.

A 10 ml culture of yeast expressing CUB-PRA1 was grown for 8 h in SD-LEU. The culture was centrifuged, cells were resuspended in 100 μl of fresh SD-LEU media, and inoculated into 100 ml of SD-LEU to grow overnight. 30 OD_546_ was isolated as a pellet, resuspended in 50 ml YPD, and transferred to 150 ml of YPD in a culture flask. The culture was grown to an OD_546_ of 0.6 and divided into four 50 ml conical tubes, centrifuged, and resuspended in 30 ml water and centrifuged again to isolate a pellet. Seven micrograms of the mouse neuronal cDNA library was transformed into each of the four conical tubes. Transformed cells were centrifuged again after transformation and resuspended in 3 ml YPD. All four tubes were pooled into one and allowed to recover at 30 °C for 1.5 h in a shaker. Cells were then centrifuged and resuspended in a 4.8 ml saline solution and plated on 150 mm SD–TRP–LEU–HIS–ADE media. The plates were incubated at 30 °C for 4 days. This screening protocol was run twice.

After the screen was completed, individual yeast colonies were isolated, inoculated in liquid media, lysed using acid-washed glass beads, and a plasmid mix was purified using Qiagen Miniprep kit spin columns (Hilden, Germany). This plasmid cocktail was re-transformed into competent *E. coli*. The bait vector contains a Kanamycin resistance marker, while the library vector contains an Ampicillin resistance marker. To specifically isolate library vectors, transformed cells were plated on LB plates that contained Carbenicillin (GoldBio). Two colonies from each transformation were grown and plasmids were cut with the *Sfi*I restriction enzyme to confirm that both contain the same size insert. The isolated constructs were then re-transformed into a fresh culture of the NMY51 yeast strain carrying the bait vector and plated on dropout media to confirm the initial hit. The library prey vectors were then sequenced using the primer suggested by Dualsystems Biotech: 5′ GTCGAAAATTCAAGACAAGG 3′. The resulting sequence was run through the BLASTX algorithm to identify the isolated cDNA. Multiple genes were independently isolated more than once during the screen, suggesting that saturation was reached (Table 1).

#### Bi-molecular fluorescence complementation (BiFC) assay

We further confirmed three interactions using the BiFC assay in mammalian cells. ζ1-COP, LAMTOR2, and Calmodulin-3 were chosen since we were able to detect that their library vectors contained full-length open reading frames after sequencing. The BiFC vectors pBiFC-VN173 (Addgene plasmid #22010) and pBiFC-VC155 (Addgene plasmid #22011) were used in this study [18]. PRA1 was cloned into the pBiFC-VN173 vector using the *Eco*RI and *Bgl*II restriction enzyme sites in frame with the downstream N-terminal Venus fragment. ζ1-COP, LAMTOR2, and Calmodulin-3 were amplified from cDNA isolated in the yeast two-hybrid screen described in this study and cloned into the pBiFC-VC155 vector using the *Eco*RI and *Bgl*II restriction sites in frame with the downstream C-terminal Venus fragment. Lamin-A, a scaffolding protein of the nuclear envelope, was selected as a negative control. It was amplified from Addgene plasmid #17662 and cloned into pBiFC-VC155. All constructs were sequence verified using Sanger sequencing by Eurofins (Louisville, KY). Constructs were delivered to cells via lipofection using Lipofectamine 2000 (Cat# 11668027), which was purchased from Life Technologies (Carlsbad, CA). COS-7 cells [CRL-1651] were purchased from ATCC (Manassas, VA). Images were acquired using an Olympus FV1000 confocal microscope (60× oil objective) 48 h after transfection.

### Results

**Fig. 1:**
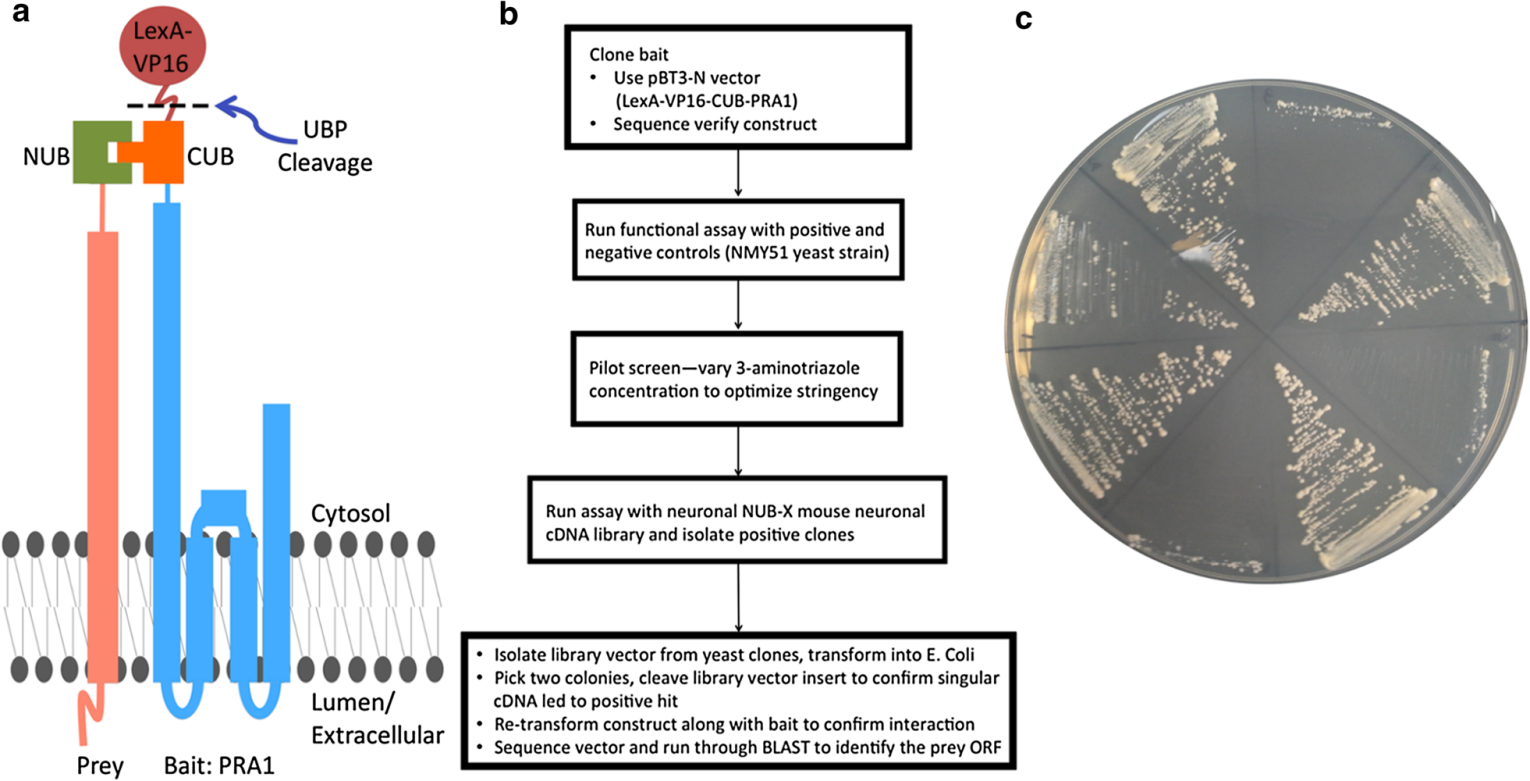
Split ubiquitin yeast two-hybrid mechanism and screening strategy. **a** LexA-VP16-CUB-PRA1 was used as bait in yeast to screen against NUB-X mouse neuronal cDNA library. An interaction leads to reconstitution of Ubiquitin and cleavage of LexA-VP16 by ubiquitin specific proteases (UBPs), which migrates to the nucleus to transcribe target genes. **b** Summary of yeast-two hybrid screening strategy. **c** PRA1 interaction with (clockwise from the 9 o’clock position): Mcm3ap, Ppargc1a, Appl1, Top1, Chmp2b, Calm3, Atpase6, and Rangrf leads to growth on dropout media (SD–LEU–HIS–TRP 10 mM 3-aminotriazole). *CUB* amino acids 34–76 of ubiquitin, *NUB* amino acids 1–38 of ubiquitin

We completed a split-ubiquitin yeast two-hybrid screen to identify PRA1 interacting partners. In this variant of the classic screening strategy, the bait (PRA1) is fused to one half of ubiquitin and a transcriptional activator. A cDNA library is then fused to the other half of ubiquitin. Interaction of the bait and prey results in the reconstitution of ubiquitin and recruitment of proteases that cleave the transcriptional activator, which then migrates to the nucleus alone (Fig. 1a) [19]. This strategy allows for the detection of an interaction of membrane proteins in their native environment and can also detect an interaction if either or both interacting proteins are integral membrane proteins. Using this screening protocol, we identified 41 novel PRA1 binding proteins (Fig. 1b, c and Table 1). Both membrane and cytosolic proteins were isolated in this screen and their annotated localization within mammalian cells suggest that they are distributed throughout the secretory pathway.

**Table 1:** Proteins found to interact with PRA1 in a split-ubiquitin yeast two-hybrid screen

Accession number	Gene name	Cellular localization	Individual isolations
NM_019434.3	Mcm3ap	Nucleus, cytoplasm	1
NM_008904.2	Ppargc1a	Nucleus, cytoplasm	3
MF113398.1	Appl1	Endosome, nucleus	1
NM_009408.2	Top1	Nucleus	1
NM_026879.3	Chmp2b	Endosome, cytoplasm	1
NM_007590.3	Calm3	Cytoplasm, microtubules	3
AF093677.1	Atpase6	Mitochondria	1
NM_021329.3	Rangrf	Plasma membrane, nucleus, cytoplasm	1
NM_001359645.1	Rab3ip	Nucleus, cytoplasm, actin bundles	1
AC160456.8	Fam155a	Membrane	1
NM_031248.4	Lamtor2	Lysosome, endosome	1
NM_172516.4	Dstyk	Plasma membrane	1
NM_010500.2	Ier5	Nucleus, cytoplasm	1
NM_011343.3	Sec61g	ER	1
XM_011241332.1	Mgll	Cytoplasm, membrane	1
U93702.1	Neu1	Lysosome, plasma membrane	1
NR_152859.1	Dmtf1	Nucleus	1
NM_001347498.1	Unc5d	Plasma membrane	1
NM_022656.2	Nisch	Endosome, plasma membrane	1
NM_001326585.1	Rhno1	Nucleus	3
NM_020045.3	Hirip5	Cytoplasm, mitochondria	1
NM_145380.2	Eif3m	Cytoplasm	1
NM_023831.3	Ift46	Cilium	1
NM_026353.4	Slc48a1	Endosome, lysosome	1
EU007907.1	Alox15	Membrane, cytoplasm, lipid droplets	1
AY772010.3	Huwe1	Nucleus	1
NM_001359902.1	Ghitm	Mitochondria	1
NM_029985.2	Lrrc42	Nucleus	1
NM_001282040.1	Pmm1	Cytoplasm	1
NM_010638.4	Klf9	Nucleus	1
NM_019817.2	Copz1	Golgi, ER	1
NM_013608.3	Naca	Cytoplasm, nucleus	2
NM_017399.5	Fabp1	Cytoplasm	2
NM_020271.3	Pdxp	Cytoplasm, actin bundles	1
NM_178405.3	Atp1a2	Plasma membrane	1
NM_011356.4	Frzb	Extracellular	1
NM_019422.3	Elovl1	ER	1
NM_178119.3	Agap1	Endosome, lysosome, Golgi	1
NM_026444.4	Cs	Mitochondria	1
NM_008410.3	Itm2b	Golgi, endosome, extracellular	1
NM_001098231.1	Pdp1	Mitochondria	1

The cellular localization listed is gleaned from the UniprotKB database. Individual isolations column refers to the number of times a gene was independently identified during the screen

**Fig. 2:**
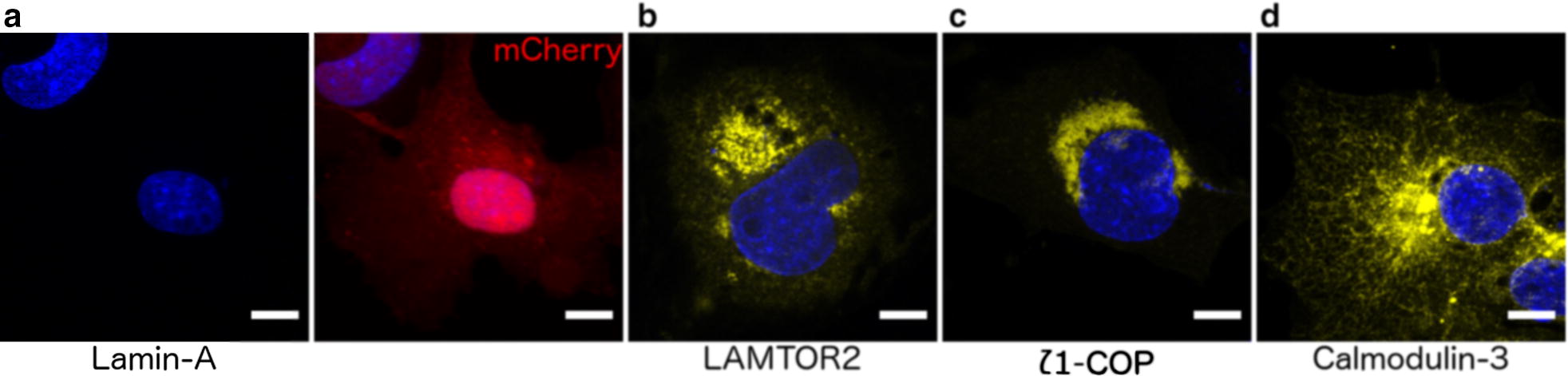
BiFC assay in COS-7 cells confirms three interactions revealed by the split-ubiquitin yeast two-hybrid screen. PRA1-VN173 was co-transfected with **a** Lamin-A-VC155 (left) and an mCherry transfection control construct (right), **b** LAMTOR2-VC155, **c** ζ1-COP-VC155, and **d** Calmodulin-3-VC155. All three identified potential interacting proteins lead to fluorescence while the Lamin-A negative control did not. Blue: DAPI, Red: mCherry, Yellow: Venus, Scale bar: 10 μm

To further validate the results of this screen, we confirmed three interactions using the BiFC assay in mammalian cells (Fig. 2). In this assay, PRA1 was fused to one half of the GFP variant Venus (VN173) and three putative PRA1 interacting proteins, ζ1-COP, LAMTOR2, and Calmodulin-3 were fused to the other half of Venus (VC155). ζ1-COP is a member of the COPI coat complex and is prevalent within the early secretory pathway, LAMTOR2 localizes to the endosomal system, and Calmodulin-3 is a cytosolic protein [20, 21]. We found that all of these proteins interact with PRA1, while Lamin-A, selected as a negative control, does not (Fig. 2).

### Discussion

The *rd1* mouse is a well characterized model of early onset rod photoreceptor degeneration. The degeneration results from a mutation in *Phosphodiesterase 6b* (*Pde6b*), which is also mutated in some cases of the human disease retinitis pigmentosa. *Pde6b* is necessary for phototransduction, but the specific molecular pathway leading to cell death is incomplete. We found that *PRA1* is the only gene that is significantly and consistently down-regulated between P2 and P8, prior to the onset of photoreceptor degeneration at P10, other than *Pde6b* itself [9]. PRA1 was initially characterized as regulator of Rab GTPase trafficking [6, 7], although further studies point to other functions within the early secretory pathway that remain to be elucidated [10]. To shed light on its true function(s) and to better understand the photoreceptor degeneration phenotype we observed in the *rd1* retina, we screened for novel binding partners using a neuronal cDNA library. Utilizing a variant of the classic yeast two-hybrid strategy and taking into account the membrane association of PRA1, we isolated 41 novel binding partners and further confirmed three interactions using a BiFC assay in cultured mammalian cells.

Of particular interest within the list of novel binding partners are those that reside in the early secretory pathway. ζ1-COP, a member of the COPI coat that facilitates retrograde trafficking from the Golgi to the ER, interacts with PRA1. This is consistent with data demonstrating that a member of the PRA1 family in *Arabidopsis* co-localizes with another member of the COPI coat, γ-COP, and that PRA1 plays a role in the anterograde trafficking of proteins at the ER [22]. Further support comes from a large-scale human protein interaction dataset that showing that PRA1 interacts with many other ER residents [23]. In mammals, PRA1 knockdown has been found to affect both COPI and COPII coat staining, further suggesting a role for PRA1 within the early secretory pathway [10]. Other studies in both plant and mammalian cells demonstrate that flux in PRA1 expression affects the rate of transmembrane protein movement through the secretory pathway [24, 25].

PRA1, as the name suggests, is a proposed Prenylated Rab Acceptor. Why did we not find any members of this ubiquitous family within our list of binding partners? We used a library with N-terminal fusion tags, which should not hinder C-terminal Rab GTPase prenylation. Of note is the fact that although PRA1 is a proposed regulator of Rab GTPase trafficking, deletion of the *PRA1* gene does not affect Rab GTPase localization [11, 12]. Furthermore, PRA1 is not just promiscuous in its interactions within the Rab GTPase family of proteins, but also with a variety of other prenylated proteins [4, 5]. Figueroa et al. [5] found that the simple addition of a CaaX box to the C-terminus of the GFP open reading frame leads to its interaction with PRA1. Direct evidence linking PRA1 to Rab GTPase trafficking has been limited to in vitro studies [7, 8, 26]. These reports suggest that the interaction between PRA1 and Rab GTPases relies on the lipid tail itself, and may not be specific in nature. Interestingly, none of the potential PRA1 binding partners identified by this study have been found to be prenylated. Together with our data, this indicates that the relationship between PRA1 and prenylated proteins requires further study to determine whether a functional output of these previously documented interactions occurs in vivo. Our results add to a growing body of literature suggesting additional role(s) for PRA1 warrant further investigation.

## Limitations

- Some interactions may be missed due to specific use of neuronal cDNA library.
- Transient, but nevertheless important interactions, may not be observed.
- N-terminal tag fused to cDNA library may disrupt protein folding and membrane protein insertion.
- Lack of complexity in yeast compared to mammalian organisms may lead to the loss of some interactions.

## Abbreviations

PRA1: Prenylated Rab Acceptor 1
BiFC: bimolecular fluorescence complementation assay
Pde6b: Phosphodiesterase 6 beta
VN173: Venus amino acids 1–172
VC155: Venus amino acids 155–238
P2: postnatal day 2
CUB: amino acids 34–76 of ubiquitin
NUB: amino acids 1–38 of ubiquitin
